# Interpretable machine learning for accessible dysphagia screening and staging in older adults

**DOI:** 10.1016/j.isci.2025.114451

**Published:** 2025-12-16

**Authors:** Yinuo Dai, Jianzheng Cai, Zhina Gong, Chunyan Niu, Weixia Yu, Haifang Wang, Yingying Zhang

**Affiliations:** 1 Department of Radiotherapy, the First Affiliated Hospital of Soochow University, Suzhou, China; 2 Department of Nursing, the First Affiliated Hospital of Soochow University, Suzhou, China

**Keywords:** Gastroenterology, Health sciences, Internal medicine, Medical specialty, Medicine

## Abstract

Dysphagia in older adults causes serious complications, and efficient and scalable screenings are needed. This prospective multicenter study developed interpretable machine learning (ML) models for the early identification and staging of dysphagia. Nine ML models were built using the clinical data from 1,235 patients and externally validated on 720 patients. All patients were older adults from seven Suzhou hospitals whose dysphagia was confirmed via videofluoroscopic swallowing studies. Features were selected via random forest, and model interpretability was analyzed with SHapley Additive exPlanations (SHAP). The CatBoost model achieved an area under the receiver operating characteristic curve (AUC) of 0.914 for binary classification, while neural network gave AUC 0.884 for multiclass classification. External validation confirmed robustness (binary AUC, 0.909 and multiclass macro-AUC, 0.860). SHAP identified ten core features—oral/pharyngeal function influenced all stages, and masticatory/phonatory features acted selectively. A web application was created accordingly to facilitate real-time screening and stratify dysphagia patients.

## Highlights

• Nine machine learning models were compared in triaging geriatric dysphagia
• The models used multi-domain data and were interpretable
• Both the binary and multiclass classifiers relied on only ten core features
• A web app with SHAP visualizations was developed to support clinical screening

## Graphical abstract

**Figure:**
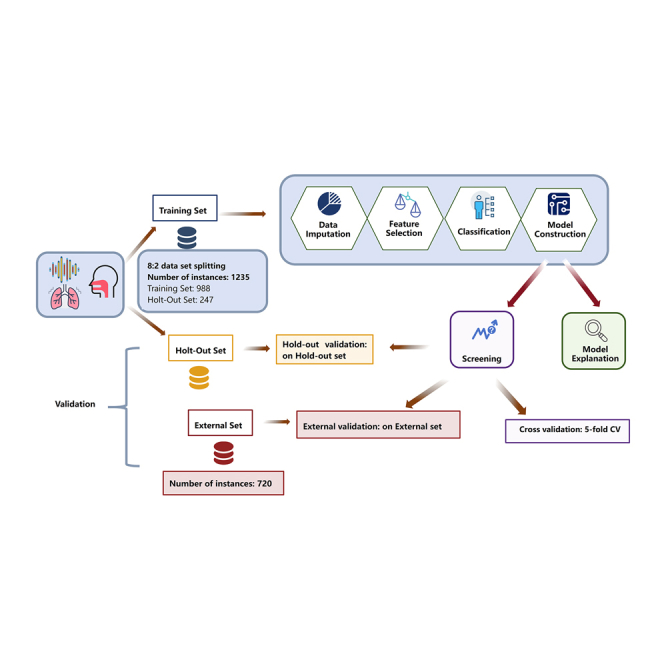

## Introduction

Dysphagia, or difficulty swallowing, is a common condition affecting 11%–33% of older adults.^1^ It interferes with the safe and effective intake of food and liquids and may lead to malnutrition and dehydration, as patients frequently restrict intake due to fear of choking or aspiration. Many withdraw from eating-related social gatherings to avoid embarrassment.^2^ In this way, nutritional challenges carry profound psychological consequences. Over time, such avoidance erodes quality of life and contributes to anxiety, depression, and emotional distress. Clinically, dysphagia can lead to serious complications, including aspiration pneumonia and elevated risks of morbidity and mortality, particularly in individuals with neurological conditions.^3^ The combined nutritional, psychological, and medical burdens place substantial strain on healthcare systems and caregivers, and effective screening and intervention are urgently needed.

The water swallowing test is a widely used method for the early screening and diagnosis of dysphagia, but it is subjective and poorly sensitive to early-stage abnormalities or phase-specific classification.^4^ More accurate tools such as videofluoroscopic swallowing studies (VFSSs) and fiberoptic endoscopic evaluation of swallowing (FEES) offer detailed assessments, but they may be inaccessible in primary care settings as they require specialized equipment and trained personnel.^5^^,^^6^ Furthermore, older adults often have comorbidities and atypical symptoms that obscure the clinical presentation of dysphagia and thus complicate accurate screening and staging.

Dysphagia involves interconnected impairments in swallowing, respiration, and phonation. It is not a single functional deficit but reflects a complex, neurally regulated condition governed by coordinated muscle activity.^7^ These coordinating muscles help maintain trunk and head posture while modulating the pharyngolaryngeal passage, thereby supporting respiration, phonation, and safe swallowing.^8^^,^^9^ Swallowing and respiration are closely linked, as both are regulated by medullary centers and operate through common neural pathways.^10^ Safe swallowing relies on precise respiratory coordination: laryngeal elevation, glottal closure, and a brief apnea (0.5–1.0 s) prevent aspiration during bolus transit.^11^^,^^12^ Swallowing and phonation are also linked through shared neuromuscular control.^13^ The laryngeal muscles that facilitate airway protection during swallowing contribute to vocalization, and many individuals with dysphagia exhibit articulation difficulties, including increased hoarseness, vocal tension and weakness, and reduced vocal fold stability.^14^^,^^15^^,^^16^ Thus, voice quality serves as a sensitive marker of swallowing dysfunction, and vocal changes are considered valuable for the early diagnosis and targeted intervention of dysphagia.^17^ The timely identification of dysphagia and its stages, particularly in older adults with comorbidities, requires multi-dimensional, efficient, and clinically precise screening approaches.

Machine learning (ML) is a powerful complement to traditional statistical methods and is now widely used in disease prediction, screening, and diagnosis.^18^ However, most ML studies in dysphagia focus on patients with specific conditions or rely on single-domain indicators.^19^^,^^20^^,^^21^ Recent works tackle one complementary dimension at a time—clinical indicators, swallowing acoustics, or respiratory signals. For example, Gugatschka et al.^22^ developed an ML-based dysphagia risk prediction tool using only clinical and demographic indicators to provide binary risk classification without subtyping. Kimura et al.^23^ applied shallow and ensemble learning to the automated detection of swallowing sounds in a clinical database (*n* = 74) and demonstrated the sensitivity of acoustic markers to swallowing alterations. Kadono and Noguchi^24^ used millimeter-wave radar and an ML method to measure respiratory movements during swallowing. Despite progress across these complementary domains, few ML studies integrate clinical, acoustic, and respiratory features to explore swallowing difficulties and their staging.^25^

Beyond data integration challenges, three key issues further hinder the clinical utility of ML-based dysphagia tools. First, in the field of dysphagia research, rigorous validation using external prospective datasets remains remarkably scarce; most studies rely solely on internal cohorts or cross-validation, which calls into question their generalizability.^26^^,^^27^ Second, conventional ML models are commonly perceived as “black boxes” because the rationale behind model decisions is typically opaque, and clinicians often struggle to trust and apply model predictions.^28^ Third, most existing models do not incorporate dysphagia staging and prove to be inadequate for precision management and personalized care. Moreover, technical barriers complicate integration into geriatric care workflows, especially when the user interface is difficult to navigate or the tool is not well-adapted to clinical settings.

Addressing these limitations requires models that provide transparent reasoning, account for clinical nuance, and can be effectively integrated into diverse care environments. Therefore, this study proposes an interpretable ML-based screening framework to identify dysphagia-affected patients and classify disease stage. To facilitate adoption in geriatric care settings, we also developed a web application that can be easily deployed in various clinical environments. By enabling early detection and stratified intervention, the system expedites more personalized care for elderly individuals with dysphagia.

## Results

### Patients

The model development and internal validation involved 1,235 patients, with 988 patients in the training set and 247 patients in the internal validation set. Of these patients, 621 (50.3%) had dysphagia and 614 (49.7%) did not.

Table S1 shows that the dysphagia group had a slightly higher age (74.34 ± 7.61 vs. 73.40 ± 6.91), higher percentage of female patients (61.19% vs. 46.42%), lower BMI (22.59 ± 3.59 vs. 23.13 ± 3.43), as well as higher rates of unrepaired tooth loss (71.98% vs. 56.84%), stroke (59.42% vs. 43.81%), Parkinson’s disease (3.70% vs. 0.33%), coronary heart disease (17.39% vs. 10.42%), and masticatory dysfunction (10.47% vs. 6.35%).

Table S2 shows that the dysphagia group had a significantly lower vital capacity (767.87 ± 553.84 mL vs. 1,346.75 ± 621.76 mL), more frequent abnormal breathing patterns during eating (e.g., oral respiration, 6.12% vs. 1.79%; tachypnea, 1.61% vs. 0.00%), and more frequent postural changes during eating (15.14% vs. 6.51%). The patients needed dietary adjustments. They scored worse in all six aspects of the Ohkuma questionnaire, and they had weaker swallowing muscles, higher GRBAS scores, and worse acoustic metrics (e.g., significantly shorter MPT).

### Feature selection

**Figure 1:**
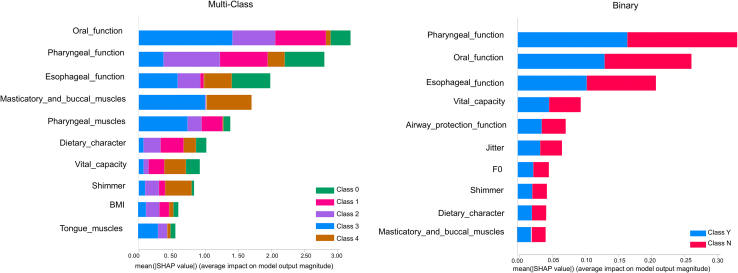
Importance of the top ten features in the (left) multiclass and (right) binary classification models Class labels: 0, no dysphagia; 1, oral phase dysphagia; 2, pharyngeal phase dysphagia; 3, esophageal phase dysphagia; 4, oropharyngeal dysphagia; N, no dysphagia; Y, dysphagia.

To develop interpretable and clinically practical ML models, we performed feature selection based on feature importance rankings derived from a Random Forest classifier. To balance model parsimony, interpretability, and clinical utility, we applied a cumulative feature importance threshold of 85% and selected the minimal set of features that exceeded this threshold. The top 10 features, identified as core predictors, accounted for 85.23% and 87.30% of the total feature importance for the multiclass and binary models, respectively (Figure 1). The selected features are listed hereafter.

1. Multiclass model: oral function, pharyngeal function, esophageal function, masticatory and buccal muscles, pharyngeal muscles, dietary character, shimmer, BMI, vital capacity, and tongue muscles.
2. Binary model: oral function, pharyngeal function, vital capacity, airway protection function, jitter, esophageal function, F0, dietary character, shimmer, and masticatory and buccal muscles.

### Model performance

**Figure 2:**
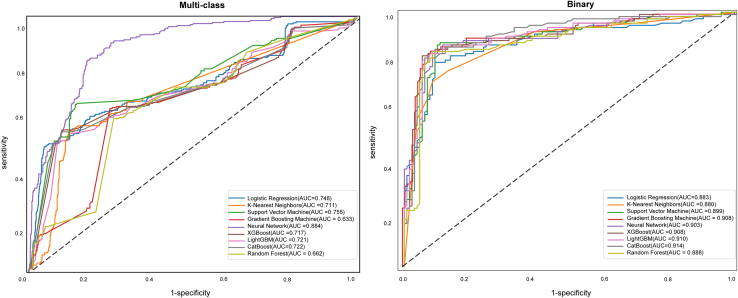
Receiver operating characteristic (ROC) curves for the machine learning models on the internal validation set AUC, area under the ROC curve.

We then applied nine algorithms for dysphagia classification, and the performance of the corresponding multiclass and binary classification models was evaluated using sensitivity, specificity, accuracy, F1 score, and receiver operating characteristic (ROC) curve analysis (Figure 2). The effectiveness of multiclass and binary classification models on training and validation sets is detailed in Tables S3–S6.

All models demonstrated strong discriminative ability. For multiclass classification, the neural network (NN) model demonstrated superior overall performance. On the internal validation set, it achieved the highest AUC of 0.884 (95% CI, 0.875–0.893), along with a sensitivity of 0.757, specificity of 0.948, accuracy of 0.845, and an F1-score of 0.838 (Figure 2, Table S5). The logistic regression (LR) and support vector machine (SVM) models showed comparable, robust performance (AUC = 0.748 and 0.755, respectively), indicating their strong capability in handling multiclass distinctions (Figure 2). In contrast, gradient boosting machine (GBM) and random forest (RF) exhibited notably lower discriminatory power, with the AUC being 0.633 and 0.662, respectively (Figure 2). Figure S2 shows the ROC curves of each model for each class of dysphagia.

For binary classification, the CatBoost model emerged as the top performer, attaining an AUC of 0.914 on the validation set (Figure 2). It also delivered balanced metrics with a sensitivity of 0.829, specificity of 0.900, accuracy of 0.880, and an F1-score of 0.866 (Table S6). The NN and LightGBM models were close competitors. Notably, LightGBM achieved the highest specificity (0.923) and tied with NN for the highest recall (0.883), making it particularly attractive for scenarios where minimizing false positives is critical (Table S6). The SVM and RF also delivered solid performance, while LR and K-nearest neighbors again trailed behind the ensemble methods.

### External validation

**Figure 3:**
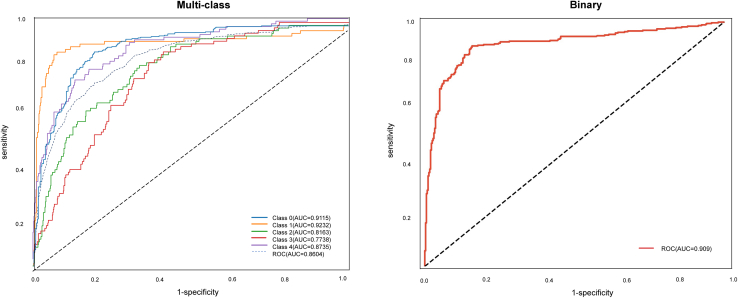
Receiver operating characteristic (ROC) curves on the external validation set Neural network model for multiclass classification (left); CatBoost model for binary classification. AUC, area under the curve (right).

External validation was performed on an independent dataset of 720 patients using the best-performing models, i.e., NN for multiclass and CatBoost for binary classification. This validation more accurately reflects the model’s clinical applicability, as it tests the model’s ability to generalize to patient populations that were not involved in the model’s training or internal validation process and were enrolled from a different period. Tables S7 and S8 describe the baseline characteristics of the patients in the external validation set compared to those in the model development cohort. The NN model was used for multiclass classification; class-specific AUCs were calculated, and the macro-averaged AUC was 0.860 (Figure 3, 95% CI, 0.841–0.879), which was slightly lower than the AUC obtained on the internal validation set (0.884, Figure 2). The CatBoost model for binary classification achieved an AUC of 0.909 (Figure 3, 95% CI, 0.885–0.931), which was also slightly lower than the AUC on the internal validation set (0.914, Figure 2). These findings demonstrate the strong generalizability and robustness of both models on unseen data. Table S9 gives the model performance in full.

### Model interpretation

**Figure 4:**
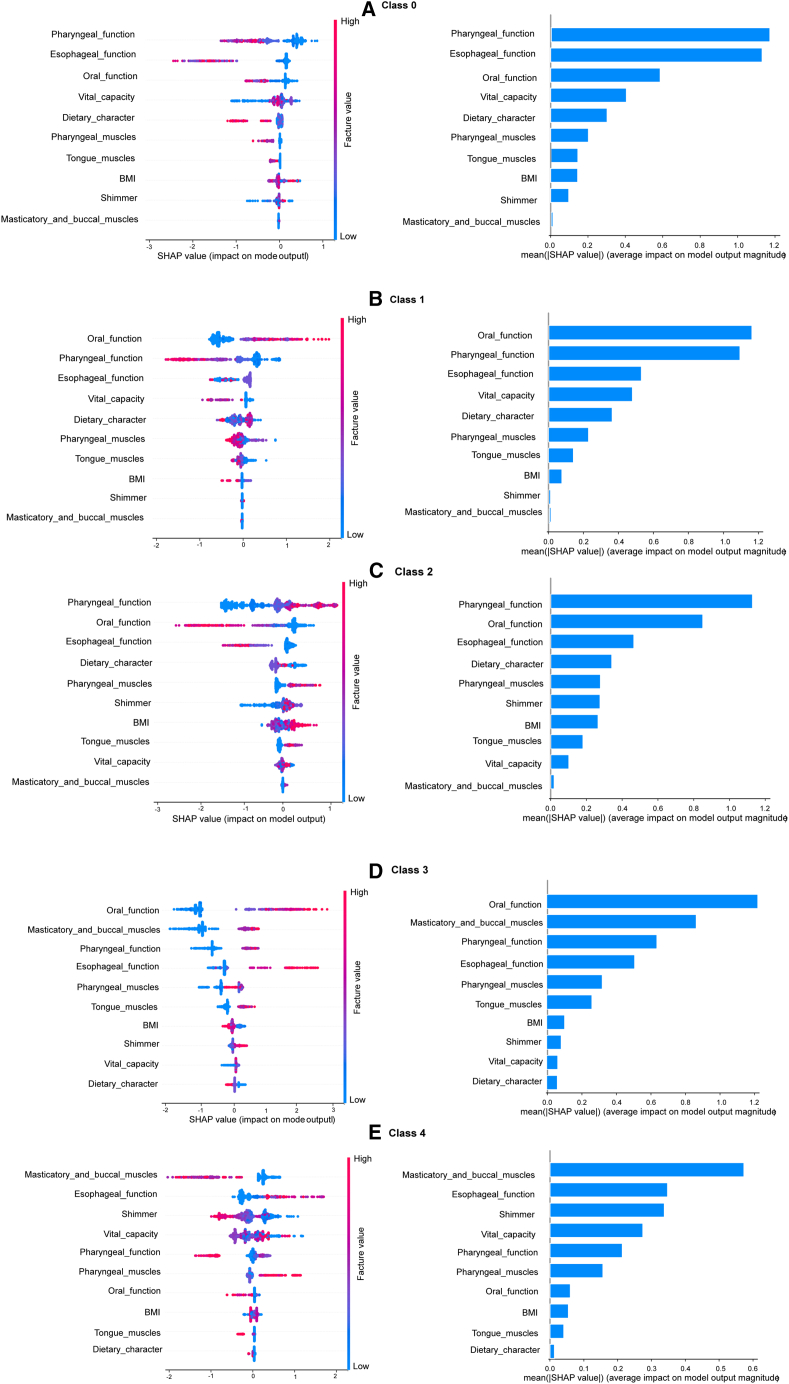
Impact of features on multiclass dysphagia prediction Images on the left side show the distribution of SHAP values for the most influential features, with red indicating high feature values and blue indicating low values. A positive SHAP value represents a positive impact on class screening. Images on the right side give the mean absolute SHAP values to demonstrate the overall importance of each feature.

**Figure 5:**
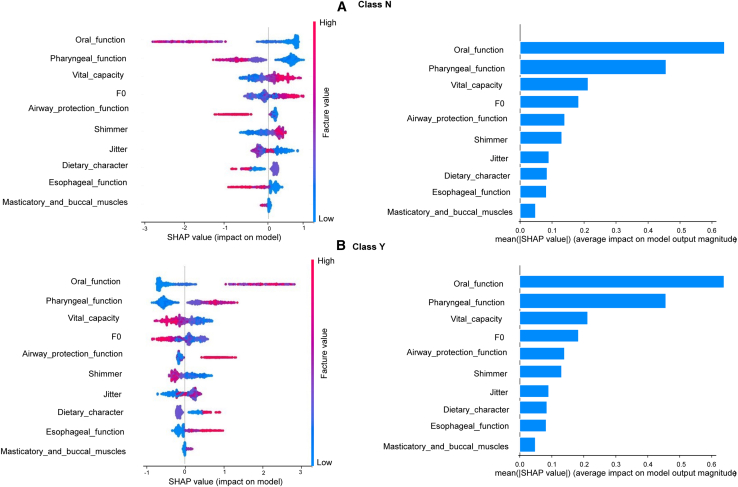
Impact of features on binary dysphagia prediction Images on the left side show the distribution of SHAP values for the most important features, with red indicating high feature values and blue indicating low values. A positive SHAP value represents a positive impact on class screening. Images on the right side give the mean absolute SHAP values to demonstrate the overall importance of each feature.

Figures 4 and 5 illustrate the contribution and distribution of key features across dysphagia outcome categories using SHapley Additive exPlanations (SHAP) plots. Each SHAP plot (left) visualizes the influence of individual features in the best-performing model, with red points representing high feature values and blue points representing low values. Features plotted further to the right exert a stronger positive influence on the model’s prediction of dysphagia categories. For example, in Figure 4D, Oral_function is the most significant feature impacting the model’s output. High values for Oral_function (represented by red points) correspond strongly with positive SHAP values, indicating that more severe oral-phase dysfunction significantly increases the model’s output probability. Conversely, low values (blue points) are associated with negative SHAP values and decreases the model’s output. A similar pattern is seen for Masticatory_and_buccal_muscles, where lower values (blue) contribute to a decreased prediction (negative SHAP) and higher values contribute to an increased prediction (positive SHAP). In contrast, for Vital_capacity, low values (blue) are associated with positive SHAP values, suggesting that a reduced Vital_capacity increases the model’s output probability.

In multiclass classification (Figure 4), oral and pharyngeal functions emerged as the most influential predictors across all outcome classes except class 4 (oropharyngeal dysphagia). Class 4 was more strongly associated with features such as esophageal function, masticatory and buccal muscle strength, vocal-fold shimmer, and vital capacity. In addition, pharyngeal muscle strength showed a broadly distributed impact across all classes. Like the multiclass model, the binary classification model (Figure 5) also relied on oral and pharyngeal functions in predicting dysphagia. However, the binary model placed greater emphasis on features such as airway protection function, F0, and jitter. That is, these features were particularly relevant in identifying the presence of dysphagia. Together, these visualizations provide insight into the underlying decision-making of the models, and they help to elucidate how specific physiological and acoustic features drive dysphagia classification.

### Deployment and use

To improve clinical accessibility and support rapid screening, we simplified model inputs to a core set of ten features. The final models were deployed as interactive web applications (Figure S2), allowing clinicians to input patient data and instantly receive classification results.

• Multiclass dysphagia classifier: https://enoch-d-multi-dysphagia.streamlit.app
• Binary dysphagia classifier: https://enoch-d-binary-dysphagia.streamlit.app

These tools provide intuitive, real-time screening for dysphagia presence and subtype. The platform presents not only the prediction but also visual explanations of how each input feature influenced the result, using SHAP-based analysis to help clinicians understand the reasoning behind each prediction.

## Discussion

Traditional dysphagia screening tools—such as the water swallow test, the volume-viscosity swallow test, and even gold-standard assessments like VFSS and FEES—are often constrained by subjectivity, resource demands, and limited accessibility. These methods require trained personnel and specialized equipment, and they are not always feasible in routine or resource-limited clinical settings. Meanwhile, bedside screening tools may lack sensitivity for detecting silent aspiration or differentiating among dysphagia subtypes. In contrast, ML-based models offer a scalable, objective, and data-driven approach to screening. By integrating multimodal physiological and acoustic features, the models can detect subtle patterns that may elude human observation, enabling earlier and more nuanced identification of dysphagia. The deployment of these models as web-based tools further enhances accessibility, allowing clinicians to perform rapid, standardized assessments without the need for specialized infrastructure.

In this work, using nine ML algorithms, we developed separate binary and multiclass classification models, each based on an optimized set of ten predictive features, to screen for dysphagia and its subtypes in elderly patients. In contrast to studies that consider an extensive list of clinical features,^22^^,^^29^ our approach only required ten features, which alleviates the burden associated with data collection. This simplification should significantly assist clinical translation, making the tool more suitable for practical use in elderly populations. The CatBoost model was the best for dysphagia detection (AUC = 0.914), whereas the NN model was the best for subtype classification (AUC = 0.884). External validation using temporally independent datasets confirmed both models’ robustness and generalizability, with the binary and multiclass models achieving AUCs of 0.909 and 0.860, respectively. Our binary classification model outperformed traditional screening tools in detecting dysphagia, reflecting ML’s growing utility across clinical domains in capturing non-linear relationships and extracting insights from complex datasets.^30^ The consistent excellence of CatBoost and NN models across both tasks underscores their suitability for modeling the complex, non-linear relationships inherent in our multi-domain clinical data. Meanwhile, the multiclass model demonstrated the capacity to differentiate dysphagia subtypes; it would thus empower targeted clinical management and informing intervention strategies tailored to specific functional deficits. By analyzing the key features contributing to each model’s predictions, we also gained insights into the physiological mechanisms underlying dysphagia.

Furthermore, we demonstrated the models’ interpretability by presenting the distribution of SHAP values for the ten screening features. In both the CatBoost model for binary classification and the NN model for subtype classification, oral and pharyngeal functions consistently emerged as dominant features, which suggests their central role in dysphagia pathophysiology. Deficits such as reduced tongue strength and impaired masticatory coordination can disrupt bolus formation and propulsion, whereas pharyngeal dysfunction elevates the risk of residue accumulation and aspiration.^31^^,^^32^ Previous studies have demonstrated that limited tongue base retraction and delayed pharyngeal swallow initiation are frequent contributors to dysphagia in older adults.^33^ To mitigate these impairments, patients often implement compensatory postural adjustments such as chin-tuck, head-turn, or head-back maneuvers to redirect bolus flow and protect the airway. Additionally, dietary modifications, including texture and viscosity adjustments, are frequently adopted to enhance swallowing safety and reduce aspiration risk.^34^^,^^35^

Vital capacity, esophageal function, dietary character, and shimmer were shared features of both models and exhibited similar SHAP magnitudes in the binary classifier. Their consistent presence suggests that they contribute broadly to dysphagia detection and subtype differentiation. Vital capacity reflects the interplay between respiratory and swallowing systems. In the present study, respiratory features centering on vital capacity demonstrated exceptional influence in predicting pharyngeal-phase dysphagia. This finding provides robust evidence for the clinical value of the respiration-swallowing coordination mechanism. Reduced pulmonary function can impair airway protection during swallowing, which increases the risk of aspiration. Studies of Parkinson’s disease and multiple sclerosis have shown that impaired respiratory function and altered breathing-swallow coordination are associated with increased aspiration risk.^36^^,^^37^^,^^38^ In a randomized controlled crossover study of patients receiving oxygen therapy, a close link is found between aspiration and altered swallowing patterns, especially when inspiration opposes the direction of the swallow.^39^ The developed ML models further enhance our understanding: The results complement the observations on apnea^24^ and elucidate the association between respiratory features and a specific subtype of dysphagia (pharyngeal-phase impairment). Esophageal function is essential for clearing the bolus and plays a key role in esophageal and oropharyngeal dysphagia. Although its SHAP value in the binary model is moderate, which suggests limited impact on detecting dysphagia overall, it proves more useful for distinguishing subtypes, likely due to the distinct underlying pathologies. Prior studies have indicated that esophageal-phase dysphagia can result from various causes, including esophageal obstruction, inflammatory changes, and primary motility disorders, as these conditions disrupt coordinated bolus transport through the esophageal lumen.^40^ Dietary character reflects behavioral responses to dysphagia, as modifying food consistency is a key compensatory strategy. Previous studies have shown that tailoring the texture and viscosity of meals improves swallow safety and helps reduce aspiration risk.^34^^,^^35^ Shimmer, an acoustic measure of amplitude perturbation, indicates instability in vocal fold vibration, an issue linked to dysfunctions such as impaired laryngeal elevation and vocal fold closure. Although these dysfunctions affect distinct physiological domains,^8^^,^^15^ they converge on shared mechanisms involving muscle tension, neurological disorders, and structural anomalies.^41^^,^^42^ As a result, compromised laryngeal control undermines safe swallowing.

Airway protection function, jitter, and F0 were features unique to the binary model. They exhibited moderate, comparable SHAP values, which highlights their relevance in distinguishing dysphagia from non-dysphagia. Airway protection function directly reflects the integrity of laryngeal closure and cough reflex, which are critical defenses against aspiration. Studies show that acoustic voice analysis can identify aspiration risk by detecting subtle post-swallow vocal changes.^41^ Jitter and F0 are voice-based acoustic markers. Elevated jitter reflects irregular vocal fold vibration, and decreased F0 suggests reduced laryngeal tension or impaired neuromuscular control. Dysfunction in jitter and F0 may signal dysphagia because the same laryngeal and pharyngeal muscles are involved in both voice and swallowing control. When the shared muscles are impaired, deficits in vocal function often coincide with compromised airway protection during swallowing. Intrinsic and extrinsic laryngeal muscles overlap functionally: the former control vocal cord tension and position, whereas the latter stabilize and mobilize the larynx during speech and swallowing.^9^^,^^43^^,^^44^ This shared muscular involvement explains why acoustic markers are valuable for binary classification but less useful for subtype differentiation. In contrast to previous studies relying solely on voice features,^45^ this work innovatively integrates acoustic markers with key clinical functions (e.g., airway protection) and respiratory features. Together, these features yield more robust screening performance than acoustic markers alone.

Pharyngeal muscles, BMI, and tongue muscles showed greater relevance for subtype differentiation, as they were retained exclusively in the multiclass model. Pharyngeal and tongue muscles participate in multiple phases of deglutition, and their strength is critical for effective swallowing. Since the deficient strength of these muscles leads to functional abnormalities such as impaired bolus propulsion or compromised airway protection, they can offer diagnostic cues for distinguishing dysphagia subtypes.^46^ BMI may serve as a proxy for nutritional status or sarcopenia, both of which are linked to the severity and subtype distribution of dysphagia in older adults.^47^ Masticatory and buccal muscle strength appeared in both models, but the SHAP values varied widely. It had high SHAP values in class 4 (oropharyngeal dysphagia) and class 3 (esophageal phase dysphagia), which suggests a strong role in characterizing these subtypes. Conversely, it had low SHAP values in class 0 (no dysphagia), class 1 (oral phase dysphagia), and the binary model, which indicates limited utility for general dysphagia detection. This variability underscores the need for class-specific SHAP interpretation, as averaged importance can obscure subtype-level contributions.

Our study offers several key advancements compared to previous ML models for dysphagia. Rather than focusing solely on swallowing-specific variables, as many prior models have done, we integrated features spanning swallowing, respiratory, and phonatory domains to better reflect the multidimensional nature of dysphagia. This comprehensive approach reduces the risk of overlooking patients with atypical or subtle symptoms—a major limitation in models restricted to feeding metrics or voice acoustics alone.^30^^,^^48^ We also addressed the interpretability challenge common in ML by applying SHAP analysis to visualize feature contributions. This not only demystifies the predictive process but also supports clinical decision-making by revealing feature-outcome associations, such as the prominence of oral and pharyngeal function across subtypes and the heightened relevance of masticatory and phonatory indicators in oropharyngeal dysphagia. To facilitate translation into practice, we deployed the models through a web-based application that distills screenings down to ten essential variables, which enables rapid, accessible screening even in primary care settings without VFSS or FEES. In doing so, our study not only enhances diagnostic precision but also aligns with broader goals of expanding access to geriatric swallowing care.

### Limitations of the study

This study has some limitations. First, the use of data exclusively from tertiary hospitals may introduce selection bias, even though we strived to enhance model reliability by rigorously validating performance through 5-fold cross-validation and multiple evaluation metrics across training, test, and external datasets. Second, the presumed utility of the web-based application in real-world clinical workflows requires further investigation, and the impact of the developed tools on patient outcomes is yet to be determined. Third, to maintain internal consistency, the initial respiratory and swallowing function assessments were conducted by a single rehabilitation specialist, and this single-assessor design at the point of care may be a limitation regarding generalizability. However, rigorous quality control measures were implemented to mitigate the risk of bias, including the use of averaged, instrument-derived readings for vital capacity and an independent video review process for swallowing evaluations. Finally, food consistency and portion size were not standardized; this strengthened real-world relevance but potentially added uncontrolled variability to swallowing parameters. Future studies could offer participants a predefined set of clinically relevant bolus textures (e.g., nectar-thick liquid and soft solid) to both improve consistency and retain choice.

## Resource availability

### Lead contact

Requests for further information and resources should be directed to and will be fulfilled by the lead contact, Yingying Zhang (13092612689@163.com).

### Materials availability

This study did not generate new unique reagents.

### Data and code availability

• Additional data may be available upon reasonable request, pending any necessary consultation with the Ethics Committee for approval. Requests to access the datasets should be directed to Yingying Zhang at 13092612689@163.com.
• We have now uploaded the fully annotated code (including data preprocessing, feature selection, model training, SHAP analysis, and web application deployment) to a publicly accessible repository at https://github.com/enoch0307/streamlitapp_cn, and the code resources will be immediately accessible to readers upon the publication of this article.
• Any other information required for reanalyzing the data reported in this article can be obtained from the lead contact upon request.

## Acknowledgments

We thank the dysphagia patients and the healthcare professionals who participated in this study for their support. We are grateful for the financial support from the 10.13039/100016078Suzhou Science and Technology Bureau Project (grant no. SKY2022120, SKY2023050, SSD2024079) and the 10.13039/100000181Scientific Research Project of Chinese Nursing Association (grant no. ZHKY202402), which has made this research possible. The funders were not involved in the study design, the collection, analysis, and interpretation of data, or the decision to submit the manuscript for publication.

## Author contributions

Y.D. and J.C. drafted the original manuscript, revised the manuscript, created visualizations, performed validation, developed software, designed methodology, conducted investigation, curated data, and conceptualized the study. Z.G. revised the manuscript, conducted investigation, and curated data. C.N. revised the manuscript, performed validation, and conducted formal analysis. W.Y. revised the manuscript, created visualizations, and curated data. H.W. revised the manuscript, provided supervision, designed methodology, and conceptualized the study. Y.Z. revised the manuscript, provided supervision, designed methodology, conceptualized the study, managed project administration, and acquired funding. All authors interpreted the data, revised the manuscript, approved the final content, and read and approved the final manuscript. All authors contributed to the article and approved the submitted version.

## Declaration of interests

The authors declare no conflicts of interest.

## STAR★Methods

### Key resources table

**Table:** 

REAGENT or RESOURCE	SOURCE	IDENTIFIER
**Deposited data**		

Dysphagia ML model code & deployment	GitHub	https://github.com/enoch0307/streamlitapp_cn

**Software and algorithms**		

Python (v3.11)	Python Software Foundation	https://www.python.org/
Shap (v0.50.0)	Python Software Foundation	https://pypi.org/project/shap/
Streamlit (v1.51)	Python Software Foundation	https://streamlit.io/
Praat (v6.1.50)	Paul Boersma and David Weenink	https://www.fon.hum.uva.nl/praat/

### Experimental model and study participant details

This study was conducted across seven tertiary hospitals in Suzhou, China, from June 2022 to October 2024, and eligible patients were enrolled continuously via convenience sampling. There were no restrictions related to race or ethnicity for patient enrollment.

A total of 1,235 patients enrolled from June 2022 to December 2023 were randomly allocated into a Training Set (*n* = 988, 80%) and an Internal Validation Set (*n* = 247, 20%) at an 8:2 ratio for model development and tuning. An additional 720 patients enrolled from January to October 2024 formed an independent External Validation Set, used solely to assess the model’s generalizability.

Sex was included as a demographic variable in the feature set. A preliminary comparison revealed a higher proportion of female patients in the dysphagia group (61.19%) compared to the non-dysphagia group (46.42%).

#### Patients were eligible if they were

1. at least 65 years old,
2. received a VFSS administered by a rehabilitation physician,
3. in a stable condition, conscious, and able to cooperate with the assessment, and
4. capable of oral intake.

#### Patients were excluded if they

1. declined to participate,
2. could not cooperate with the VFSS due to moderate-to-severe visual or auditory impairment (per WHO criteria) or cognitive impairment (defined as a Mini-Mental State Examination [MMSE] score of <24 confirmed by a neurologist),
3. had active fever or acute respiratory infection, or
4. had major systemic comorbidities, including:
  • severe heart failure (New York Heart Association [NYHA] class III–IV),
  • decompensated liver cirrhosis (Child-Pugh class C),
  • advanced chronic kidney disease (CKD stage 4–5),
  • uncontrolled malignancy,
  • acute/severe respiratory failure, or
  • other life-threatening conditions compromising swallow assessment safety or interpretation.

#### Ethics approval and consent to participate

This study was approved by the Ethics Committee of the First Affiliated Hospital of Soochow University (No. 2024271) and followed the STrengthening the Reporting of OBservational Studies in Epidemiology (STROBE) guidelines. Before enrollment, all participating patients were fully informed of the study purpose, procedures, potential risks, and benefits. Written informed consent was obtained from the participants (or their legally authorized representative) prior to any study-related assessments or data collection.

#### Consent for publication

Written informed consent for publication was obtained from all participants.

### Method details

#### Classification of dysphagia

The VFSS was performed by a rehabilitation therapist and a radiologist as follows.

1. The therapist prepared barium sulfate contrast agents in different consistencies (liquid, thick liquid, paste, solid), mixed with water as needed.
2. The patient sat upright with head erect to maintain proper posture for imaging and swallowing assessment.
3. Under the therapist’s guidance, the radiologist recorded fluoroscopic images at a rate of 30 frames per second (FPS) while assessing swallowing function. Swallowing trials were conducted in the following order: thick liquid, paste, liquid, water, and solid.
4. The team qualitatively assessed swallowing function using the Penetration-Aspiration Scale (PAS), and the observed abnormalities were documented for analysis.

Patients were classified based on VFSS findings. For binary classification, the categories were.

• N: No dysphagia
• Y: Confirmed dysphagia

For multiclass classification, five distinct phases were defined.

• 0: No dysphagia
• 1: Oral phase dysphagia
• 2: Pharyngeal phase dysphagia
• 3: Esophageal phase dysphagia
• 4: Oropharyngeal dysphagia

#### Collection of demographic information and medical history

The socio-demographic information included age, sex, and body mass index (BMI). The medically relevant information included dental conditions and comorbidities.

#### Assessments

The assessments of respiration, swallowing, and phonation were completed in 24 h after VFSS.

##### Respiration

An experienced rehabilitation physician assessed respiratory parameters according to a structured protocol. Patients first breathed naturally at rest. Vital capacity was measured using a calibrated spirometer (Vitalograph, UK), and the readings of two valid forced expiratory maneuvers were averaged. The patient was then given a standardized snack—15 g of plain soda crackers (approximately 2 pieces, 5 mm in thickness)—with 10 mL of drinking water. This snack was chosen for its easily standardized texture and compatibility with the eating habits of older adults. A trained rehabilitation physician instructed the patient to chew the entire snack and swallow it in one bolus and monitored the swallowing process throughout. If the patient failed to adhere to this instruction, their swallowing-related respiratory behavior data were excluded from analysis. The swallowing-related respiratory behavior was categorized as follows.

1. Normal pause: 0.5–1.0 s airflow cessation during swallowing.
2. Nasal/oral respiration during chewing: Primary breathing route while processing food.
3. Breathing during swallowing: Visible chest/abdominal movements mid-swallow.
4. Tachypnea: Respiratory rate >20 breaths/min or labored breathing (e.g., nasal flaring).

##### Swallowing

Swallowing muscle strength was assessed using Kubota’s Standardized Evaluation Scale for Dysphagia (1982), a validated tool grading functions from 1 (normal) to 4 (complete dysfunction). An experienced, standardized-trained rehabilitation physician assessed the following in a quiet room.

1. Tongue strength: Patient pressed tongue against hard palate/gums bilaterally; physician rated movement completion.
2. Buccal/masticatory strength:
  a. Patient inflated cheeks; physician tapped to check air leakage.
  b. Patient bit a tongue depressor with molars; physician pulled to test grip strength (both sides).
3. Pharyngeal function: Patient vocalized/ah/with mouth open; physician rated soft palate elevation/symmetry.

To ensure the objectivity and accuracy of the assessments, key components of the swallowing evaluation were videotaped by a research team member. Subsequently, these recordings were independently reviewed and confirmed by a second rehabilitation therapist who was blinded to the initial assessment results.

The patient then chose a meal on a menu and finished the food in the usual way. During consumption, the physician observed and recorded posture changes and dietary habits. Postural change (yes/no) denoted any noticeable positional shift (e.g., leaning forward, reclining, change from sitting to semi-sitting).

For further assessment, the patient completed the Ohkuma questionnaire, which covers 15 items across six domains: pneumonia history (item 1), nutritional status (item 2), pharyngeal function (items 3–7), oral function (items 8–11), esophageal function (items 12–14), and airway protection (item 15).

##### Phonation

In a quiet room (<40 dB noise), patients discussed their medical history with three physicians in a natural voice. Each physician independently rated voice quality using the GRBAS scale: grade (overall severity), roughness (irregular vibrations), breathiness (air leakage), asthenia (weakness), and strain (excessive effort). Each parameter was scored as 0 (normal), 1 (mild impairment), 2 (moderate impairment), or 3 (severe impairment). The physicians received pre-study training to ensure consistent ratings (Cohen’s kappa >0.8). The final score was the average of three ratings.

In addition, three recordings of the patient’s voice were obtained as follows. For voice recording analysis, the patient produced sustained/ah/sounds at a normal pitch. All voice recordings were acquired using the Praat software (Version 6.1.50) with a sampling frequency of 44,100 Hz. The steadiest of three recordings was analyzed using Praat to measure the maximum phonation time (MPT), fundamental frequency (F0), fundamental frequency perturbation (jitter), amplitude perturbation (shimmer), and harmonic-to-noise ratio (HNR). The specific extraction methods for each acoustic parameter are shown in Figure S3.

##### Feature collection summary

Prior to feature selection, the initial feature space comprised 37 candidate variables extracted from the following domains.

1. Demographic information and medical history (12 features): Age, sex, BMI, tooth loss status, stroke (binary), Alzheimer’s disease(binary), Parkinson’s disease (binary), amyotrophic lateral sclerosis (binary), multiple sclerosis (binary), coronary heart disease (binary), brain injury (binary), masticatory dysfunction (binary).
2. Respiratory function (2 features): Vital capacity (mL), breathing mode during swallowing.
3. Swallowing function (13 features): Tongue muscle strength, buccal/masticatory muscle strength, pharyngeal muscle strength, posture changes while eating (binary), eating position, dietary character, reduced appetite (binary), and six domains (from 15 items) of the Ohkuma questionnaire (pneumonia history, nutritional status, pharyngeal function, oral function, esophageal function, airway protection function).
4. Phonatory function (10 features): GRBAS scale scores (Grade, Roughness, Breathiness, Asthenia, Strain), maximum phonation time (MPT, s), fundamental frequency (F0, Hz), fundamental frequency perturbation (jitter, %), amplitude perturbation (shimmer, %), harmonic-to-noise ratio (HNR, dB).

### Quantification and statistical analysis

#### Model development

Feature importance was ranked using the random forest (RF) algorithm, and “core” variables were selected to build a simplified model. Nine ML algorithms were employed: LightGBM (Light Gradient Boosting Machine), GBM (Gradient Boosting Machine), XGBoost (eXtreme Gradient Boosting), LR (Logistic Regression), CatBoost (Categorical Boosting), NN (Neural Network), KNN (K-Nearest Neighbors), SVM (Support Vector Machine), and RF (Random Forest). 5-fold cross-validation was used to ensure model stability, and grid search was applied to optimize the parameters for each algorithm. Table S10 lists the range of the parameters used in the grid search. The optimal model was selected based on the AUC. Final model validation included both internal and external datasets, and the performance was evaluated using AUC, sensitivity, specificity, recall, F1 score, and accuracy.

#### Model interpretation

To enhance the interpretability of the models and overcome their “black-box” nature, we used variable importance, rank-order plots, and SHAP values (SHapley Additive Explanations) to visualize the results. This approach enabled a more intuitive understanding of each feature’s role and contribution to the screening results, thereby boosting the model’s transparency and comprehensibility. The analysis was applied to both multiclass and binary classification.

#### Clinical tool development

We built an interactive web application platform using Streamlit, an open-source Python framework. We adopted a modular development strategy to integrate the front-end interaction and back-end services of the dysphagia screening system.

Continuous variables were presented as mean ± standard deviation (SD), and the Shapiro–Wilk test was used to assess their normality. The variables were compared using independent Student’s *t* test if they were normally distributed or the Mann–Whitney U test if otherwise. Categorical variables were presented as percentage (ratio) and compared using the Pearson chi-square test. All analyses were performed in Python 3.11.9, and the threshold of statistical significance was set at a two-tailed *p*-value of 0.05.
