# Supplementary material for: Interpretable machine learning for accessible dysphagia screening and staging in older adults
Source: iScience. 2025 Dec 16;29(1):114451. doi: 10.1016/j.isci.2025.114451 (PMC12803820; doi:10.1016/j.isci.2025.114451)
Supplement: Document S1. Figures S1–S3 and Tables S1–S10 [file mmc1.pdf]

iScience, Volume *29*

Supplemental information

Interpretable machine learning for accessible dysphagia screening and staging in older adults

Yinuo Dai, Jianzheng Cai, Zhina Gong, Chunyan Niu, Weixia Yu, Haifang Wang, and Yingying Zhang

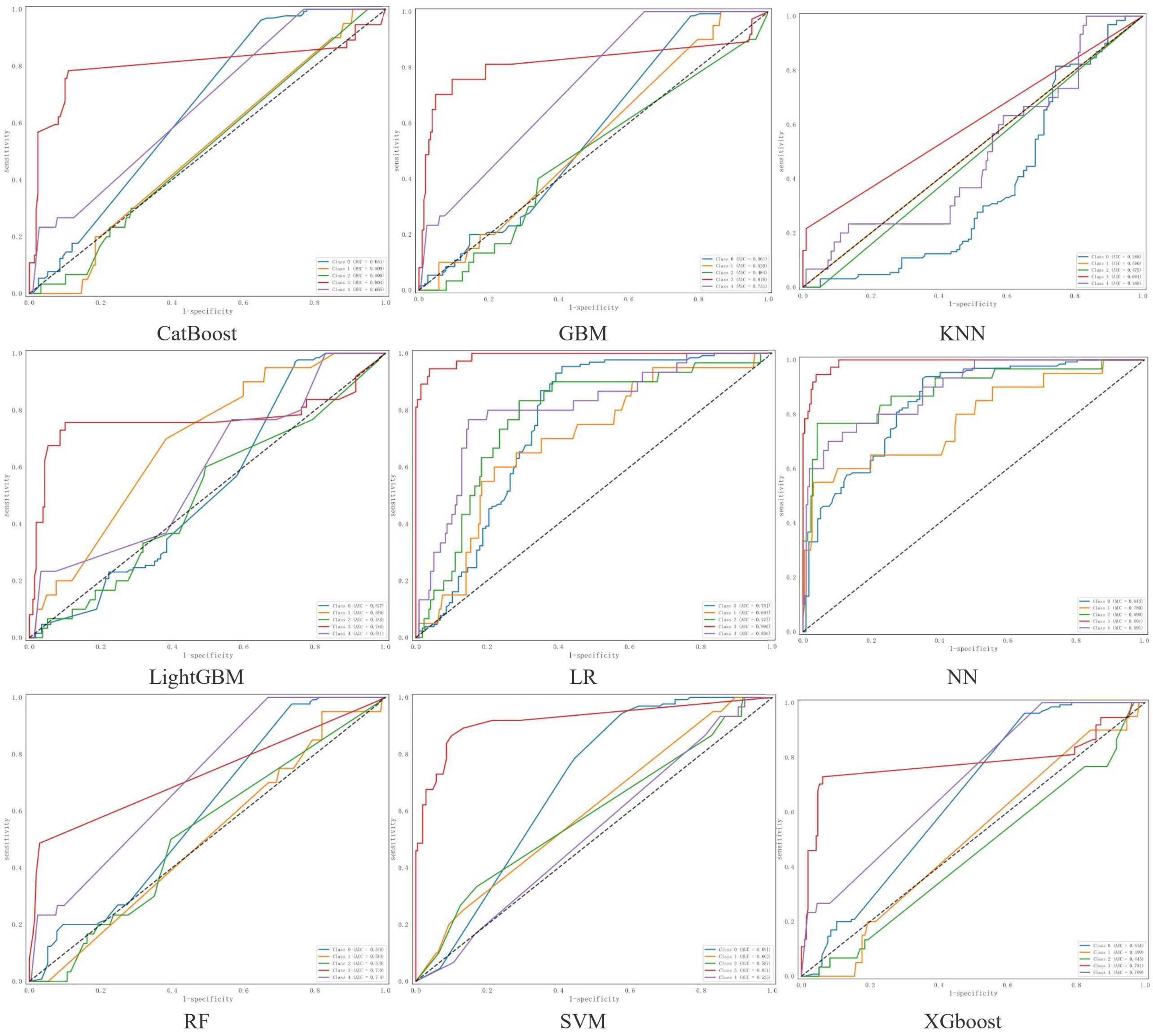

Figure S1. Multiclass screening efficiency of each model on the internal validation set. CatBoost, Categorical Boosting; GBM, Gradient Boosting Machine; KNN, K-Nearest Neighbors; LightGBM, Light Gradient Boosting Machine; LR, Logistic Regression; NN, Neural Network; RF, Random Forest; SVM, Support Vector Machine; XGBoost, eXtreme Gradient Boosting.

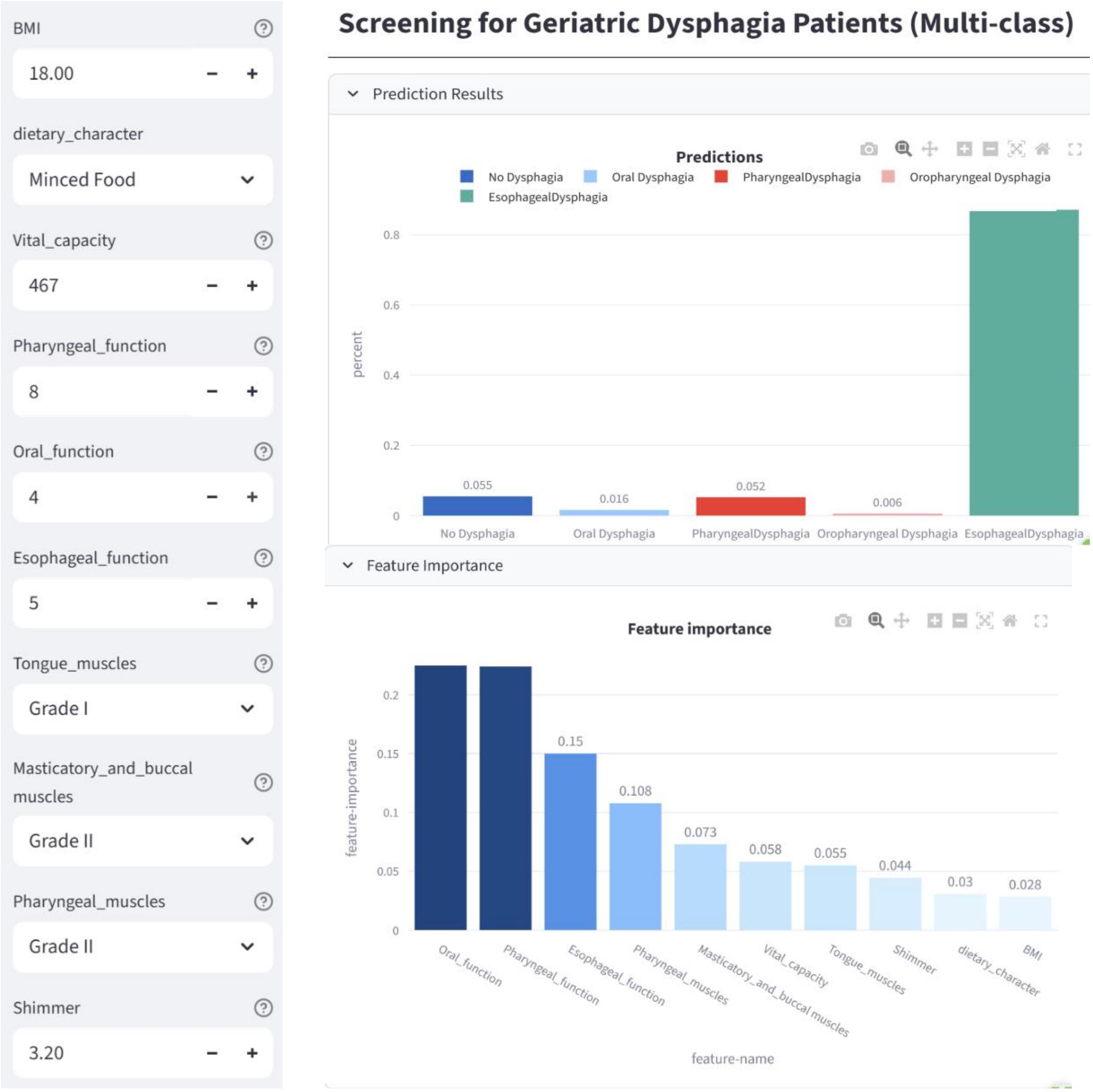

(A) Multiclass dysphagia classifier

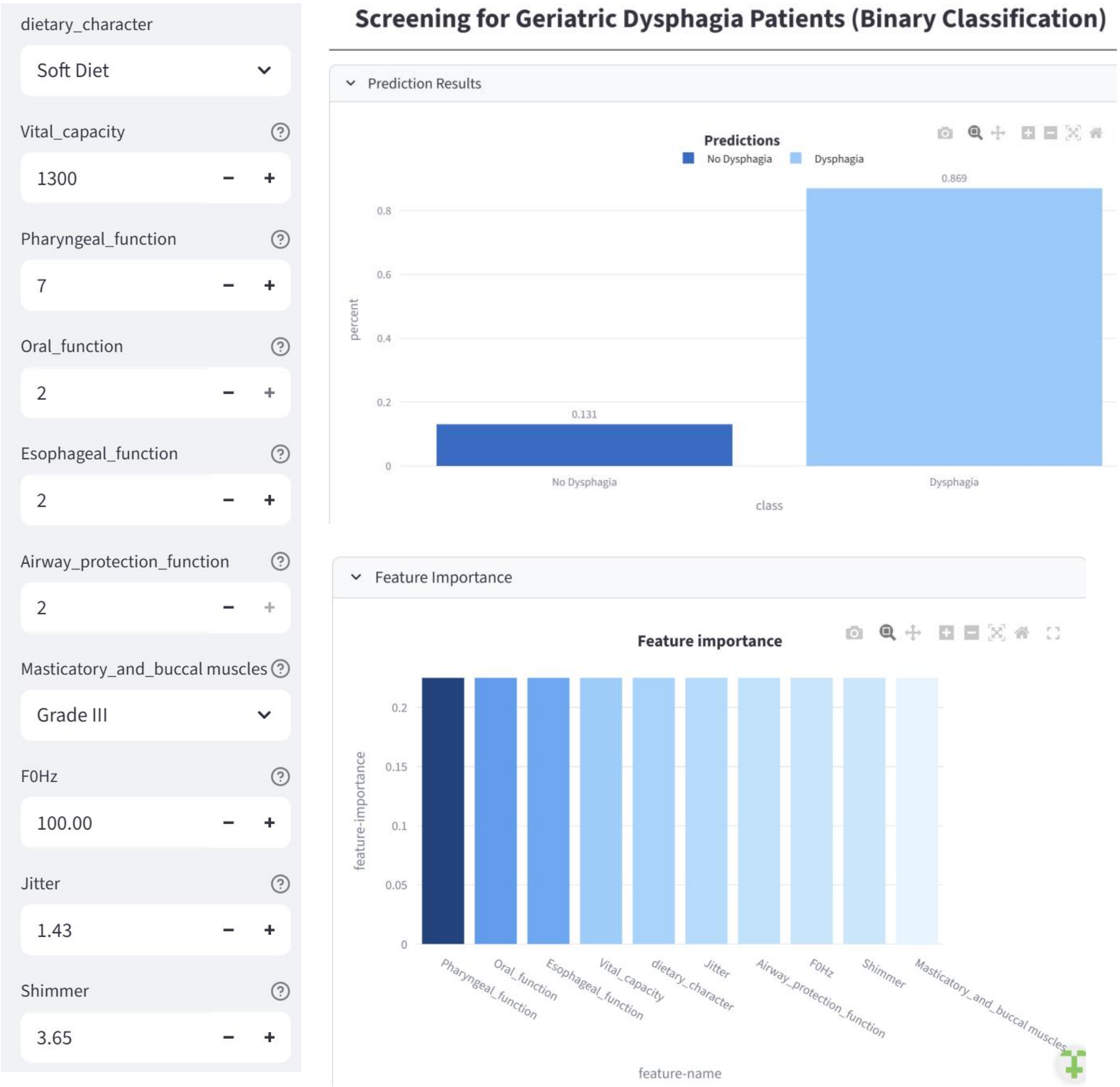

(B) Binary dysphagia classifiers

Figure S2. Interactive web applications of binary and multiclass dysphagia classifiers. Left: User input of ten core features; Top right: Screening result; Bottom right: SHAP plots explaining screening results. A positive SHAP value represents a positive impact on class screening.

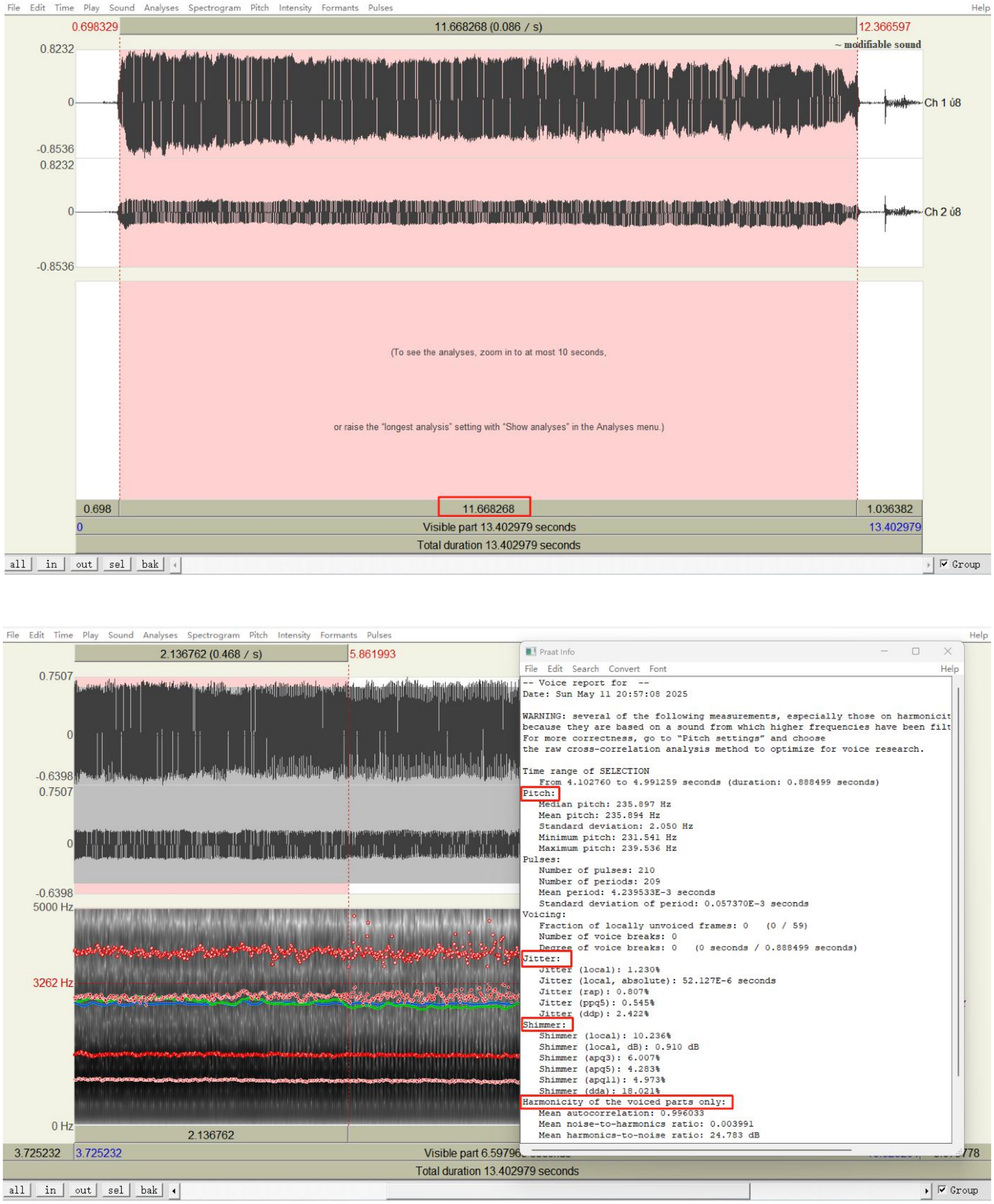

Figure S3. Extraction of acoustic parameters. Top, MPT; Bottom, F0, jitter, shimmer, and HNR.

Table S1. Patient data for model development: Demographics and medical history.

Characteristics†	All patients (n = 1235)	Non-Dysphagia (n = 614)	Dysphagia (n = 621)	***P***
Demographics				
Age (years)	73.87 ± 7.28	73.40 ± 6.91	74.34 ± 7.61	0.023
Sex				<0.001
Male	570 (46.15%)	329 (53.58%)	241 (38.81%)	
Female	665 (53.85%)	285 (46.42%)	380 (61.19%)	
BMI (kg/m^2)§	22.86 ± 3.52	23.13 ± 3.43	22.59 ± 3.59	0.007
Medical conditions				
Tooth loss				<0.001
No tooth loss	133 (10.77%)	79 (12.87%)	54 (8.70%)	
Fixed prosthesis	132 (10.69%)	90 (14.66%)	42 (6.76%)	
Removable prosthesis	127 (10.28%)	88 (14.33%)	39 (6.28%)	
Tooth loss without repair	796 (64.45%)	349 (56.84%)	447 (71.98%)	
Edentulous	47 (3.81%)	8 (1.30%)	39 (6.28%)	
Comorbidities				
Stroke	638 (51.66%)	269 (43.81%)	369 (59.42%)	<0.001
Alzheimer's disease	90 (7.29%)	37 (6.03%)	53 (8.53%)	0.090
Parkinson's disease	25 (2.02%)	2 (0.33%)	23 (3.70%)	<0.001
Amyotrophic lateral sclerosis	4 (0.32%)	0 (0.00%)	4 (0.65%)	0.044
Multiple sclerosis	15 (1.21%)	7 (1.14%)	8 (1.29%)	0.812
Coronary heart disease	172 (13.93%)	64 (10.42%)	108 (17.39%)	<0.001
Brain injury	9 (0.73%)	2 (0.33%)	7 (1.13%)	0.098
Masticatory dysfunction	104 (8.42%)	39 (6.35%)	65 (10.47%)	0.009

† Continuous variables were presented as mean ± standard deviation (SD) and compared using independent Student's t-test. Categorical variables were presented as percentage (ratio) and compared using the Pearson chi-square test. § Body mass index.

Table S2. Patient data for model development: Assessment results.

Characteristics[†]	All patients (n = 1235)	Non-Dysphagia (n = 614)	Dysphagia (n = 621)	*P*
**Respiration**				
Vital capacity (mL)	1055.67 ± 655.74	1346.75 ± 621.76	767.87 ± 553.84	<0.001
Breathing mode				<0.001
Normal pause of breath during swallowing	1074 (86.96%)	541 (88.11%)	533 (85.83%)	
Nasal respiration during chewing	65 (5.26%)	46 (7.49%)	19 (3.06%)	
Oral respiration during chewing	49 (3.97%)	11 (1.79%)	38 (6.12%)	
Breathing when swallowing	37 (3.00%)	16 (2.61%)	21 (3.38%)	
Tachypnea during eating	10 (0.81%)	0 (0.00%)	10 (1.61%)	
**Swallowing**				
Posture changes while eating				<0.001
No	1101 (89.15%)	574 (93.49%)	527 (84.86%)	
Yes	134 (10.85%)	40 (6.51%)	94 (15.14%)	
Eating position				<0.001
Sitting	299 (24.21%)	99 (16.12%)	200 (32.21%)	
Semi-sitting	927 (75.06%)	513 (83.55%)	414 (66.67%)	
Reclining	9 (0.73%)	2 (0.33%)	7 (1.13%)	
Diet				<0.001
Regular diet	334 (27.04%)	166 (27.04%)	168 (27.05%)	
Soft diet	756 (61.21%)	446 (72.64%)	310 (49.92%)	
Minced diet	36 (2.91%)	0 (0.00%)	36 (5.80%)	
Liquid diet	109 (8.83%)	2 (0.33%)	107 (17.23%)	
Reduced appetite				<0.001
No	686 (55.55%)	443 (72.15%)	243 (39.13%)	
Yes	549 (44.45%)	171 (27.85%)	378 (60.87%)	
Ohkuma questionnaire				
History of pneumonia	1.65 ± 0.67	1.47 ± 0.59	1.83 ± 0.70	<0.001
Nutritional status	1.33 ± 0.63	1.03 ± 0.18	1.63 ± 0.76	<0.001
Pharyngeal function	7.12 ± 2.37	5.64 ± 0.90	8.59 ± 2.46	<0.001
Oral function	5.21 ± 2.02	4.06 ± 0.24	6.34 ± 2.33	<0.001
Esophageal function	3.93 ± 1.83	3.07 ± 0.29	4.78 ± 2.27	<0.001
Airway protection function	1.62 ± 0.80	1.28 ± 0.52	1.96 ± 0.88	<0.001

Strength of swallowing muscles				
Tongue muscles	1.37± 0.68	1.05 ± 0.24	1.69 ± 0.81	<0.001
Masticatory and buccal muscles	1.41 ± 0.68	1.10 ± 0.29	1.72 ± 0.81	<0.001
Pharyngeal muscles	1.32 ± 0.58	1.02 ± 0.14	1.62 ± 0.69	<0.001
**Phonation**				
GRBAS				
Grade	0.43 ± 0.52	0.17 ± 0.38	0.69 ± 0.52	<0.001
Roughness	0.30 ± 0.47	0.10 ± 0.30	0.49 ± 0.52	<0.001
Breathiness	0.07 ± 0.25	0.03 ± 0.17	0.10 ± 0.31	<0.001
Asthenia	0.14 ± 0.36	0.08 ± 0.27	0.21 ± 0.42	<0.001
Strain	0.11 ± 0.31	0.04 ± 0.19	0.18 ± 0.38	<0.001
Acoustic metrics				
Maximum phonation time (MPT, s)	6.76 ± 5.20	8.36 ± 5.46	5.18 ± 4.39	<0.001
Fundamental frequency (F0, Hz)	171.80 ± 63.14	178.66 ± 46.63	165.01 ± 75.45	<0.001
Jitter (%)	0.62 ± 0.49	0.49 ± 0.41	0.75 ± 0.52	<0.001
Shimmer (%)	2.36 ± 1.52	2.22 ± 1.58	2.51 ± 1.44	0.001
Harmonic noise ratio (HNR, dB)	17.72 ± 4.472	19.00 ± 4.18	16.45 ± 4.39	<0.001

† Continuous variables were presented as mean ± standard deviation (SD) and compared using independent Student's t-test. Categorical variables were presented as percentage (ratio) and compared using the Pearson chi-square test.

Table S3. Efficacy of multiclass classification models on the training set.

Model§	Sensitivity	Specificity	Recall	Accuracy	F1 score
LR	0.788	0.949	0.828	0.828	0.827
KNN	0.785	0.952	0.855	0.855	0.850
SVM	0.818	0.955	0.851	0.851	0.851
GBM	0.826	0.960	0.877	0.877	0.874
NN	0.805	0.956	0.866	0.866	0.863
XGBoost	0.865	0.969	0.909	0.909	0.907
LightGBM	0.861	0.968	0.907	0.907	0.905
CatBoost	0.806	0.955	0.861	0.861	0.858
RF	0.880	0.972	0.920	0.920	0.919

§ LR, Logistic Regression; KNN, K-Nearest Neighbors; SVM, Support Vector Machine; GBM, Gradient Boosting Machine; NN, Neural Network; XGBoost, eXtreme Gradient Boosting; LightGBM, Light Gradient Boosting Machine; CatBoost, Categorical Boosting; RF, Random Forest.

Table S4. Efficacy of binary classification models on the training set.

Model§	Sensitivity	Specificity	Recall	Accuracy	F1 score
LR	0.758	0.931	0.889	0.839	0.889
KNN	0.792	0.931	0.857	0.857	0.864
SVM	0.809	0.929	0.865	0.865	0.900
GBM	0.872	0.946	0.907	0.907	0.910
NN	0.853	0.929	0.889	0.889	0.889
XGBoost	0.874	0.938	0.904	0.904	0.904
LightGBM	0.876	0.942	0.907	0.907	0.907
CatBoost	0.868	0.931	0.907	0.898	0.911
RF	0.885	0.963	0.922	0.922	0.922

§ LR, Logistic Regression; KNN, K-Nearest Neighbors; SVM, Support Vector Machine; GBM, Gradient Boosting Machine; NN, Neural Network; XGBoost, eXtreme Gradient Boosting; LightGBM, Light Gradient Boosting Machine; CatBoost, Categorical Boosting; RF, Random Forest.

Table S5. Efficacy of multiclass classification models on the internal validation set.

Model§	Sensitivity	Specificity	Recall	Accuracy	F1 score
LR	0.707	0.936	0.806	0.806	0.802
KNN	0.726	0.915	0.826	0.781	0.768
SVM	0.757	0.946	0.826	0.814	0.818
GBM	0.747	0.947	0.842	0.838	0.834
NN	0.757	0.948	0.845	0.845	0.838
XGBoost	0.744	0.946	0.838	0.838	0.834
LightGBM	0.757	0.947	0.842	0.842	0.832
CatBoost	0.752	0.946	0.838	0.838	0.834
RF	0.736	0.945	0.826	0.826	0.824

§ LR, Logistic Regression; KNN, K-Nearest Neighbors; SVM, Support Vector Machine; GBM, Gradient Boosting Machine; NN, Neural Network; XGBoost, eXtreme Gradient Boosting; LightGBM, Light Gradient Boosting Machine; CatBoost, Categorical Boosting; RF, Random Forest.

Table S6. Efficacy of binary classification models on the internal validation set.

Model§	Sensitivity	Specificity	Recall	Accuracy	F1 score
LR	0.735	0.915	0.830	0.834	0.828
KNN	0.726	0.915	0.826	0.834	0.824
SVM	0.803	0.908	0.858	0.860	0.858
GBM	0.846	0.892	0.870	0.885	0.870
NN	0.855	0.908	0.883	0.867	0.882
XGBoost	0.838	0.900	0.870	0.888	0.870
LightGBM	0.838	0.923	0.883	0.884	0.882
CatBoost	0.829	0.900	0.866	0.880	0.866
RF	0.838	0.915	0.879	0.878	0.878

§ LR, Logistic Regression; KNN, K-Nearest Neighbors; SVM, Support Vector Machine; GBM, Gradient Boosting Machine; NN, Neural Network; XGBoost, eXtreme Gradient Boosting; LightGBM, Light Gradient Boosting Machine; CatBoost, Categorical Boosting; RF, Random Forest.

Table S7. Baseline characteristics of the model development cohort and the external validation cohort: Demographics and medical history.

Characteristics[†]	Model development (n = 1235)	External validation (n = 720)	***P***
Demographics			
Age (years)	73.87 ± 7.28	74.82 ± 7.23	0.005
Sex			<0.001
Male	570 (46.15%)	302 (41.94%)	
Female	665 (53.85%)	418 (58.06%)	
BMI (kg/m^2)[§]	22.86 ± 3.52	22.91 ± 3.34	0.749
Medical conditions			
Tooth loss			<0.001
No tooth loss	133 (10.77%)	71 (9.86%)	
Fixed prosthesis	132 (10.69%)	56 (7.78%)	
Removable prosthesis	127 (10.28%)	70 (9.72%)	
Tooth loss without repair	796 (64.45%)	436 (60.56%)	
Edentulous	47 (3.81%)	87 (12.08%)	
Comorbidities			
Stroke	638 (51.66%)	417 (57.92%)	0.007
Alzheimer's disease	90 (7.29%)	48 (6.67%)	0.605
Parkinson's disease	25 (2.02%)	13 (1.81%)	0.735
Amyotrophic lateral sclerosis	4 (0.32%)	3 (0.42%)	0.740
Multiple sclerosis	15 (1.21%)	7 (0.97%)	0.624
Coronary heart disease	172 (13.93%)	92 (12.78%)	0.473
Brain injury	9 (0.73%)	3 (0.42%)	0.394
Masticatory dysfunction	104 (8.42%)	78 (10.83%)	0.077

[†] Continuous variables were presented as mean ± standard deviation (SD) and compared using independent Student's t-test. Categorical variables were presented as percentage (ratio) and compared using the Pearson chi-square test. [§] Body mass index.

Table S8. Baseline characteristics of the model development cohort and the external validation cohort: Assessment results.

Characteristics†	Model development (n = 1235)	External validation (n = 720)	*P*
**Respiration**			
Vital capacity (mL)	1055.67 ± 655.74	1112.71 ± 680.37	0.070
Breathing mode			<0.001
Normal pause of breath during swallowing	1074 (86.96%)	667 (92.64%)	
Nasal respiration during chewing	65 (5.26%)	20 (2.78%)	
Oral respiration during chewing	49 (3.97%)	8 (1.11%)	
Breathing when swallowing	37 (3.00%)	21 (2.92%)	
Tachypnea during eating	10 (0.81%)	4 (0.56%)	
**Swallowing**			
Posture changes while eating			0.431
No	1101 (89.15%)	650 (90.28%)	
Yes	134 (10.85%)	70 (9.72%)	
Eating position			<0.001
Sitting	299 (24.21%)	521 (72.36%)	
Semi-sitting	927 (75.06%)	184 (25.56%)	
Reclining	9 (0.73%)	15 (2.08%)	
Diet			0.001
Regular diet	334 (27.04%)	143 (19.86%)	
Soft diet	756 (61.21%)	505 (70.14%)	
Minced diet	36 (2.91%)	13 (1.81%)	
Liquid diet	109 (8.83%)	59 (8.19%)	
Reduced appetite			0.031
No	686 (55.55%)	436 (60.56%)	
Yes	549 (44.45%)	284 (39.44%)	
Ohkuma questionnaire			
History of pneumonia	1.65 ± 0.67	1.52 ± 0.65	<0.001
Nutritional status	1.33 ± 0.63	1.27 ± 0.53	0.018
Pharyngeal function	7.12 ± 2.37	6.71 ± 1.83	<0.001
Oral function	5.21 ± 2.02	5.05 ± 1.70	0.069

Esophageal function	3.93 ± 1.83	3.48 ± 0.98	<0.001
Airway protection function	1.62 ± 0.80	1.61 ± 0.79	0.882
Strength of swallowing muscles			
Tongue muscles	1.37± 0.68	1.28 ± 0.54	0.001
Masticatory and buccal muscles	1.41 ± 0.68	1.30 ± 0.56	<0.001
Pharyngeal muscles	1.32 ± 0.58	1.17 ± 0.42	<0.001
**Phonation**			
GRBAS			
Grade	0.43 ± 0.52	0.40 ± 0.53	0.183
Roughness	0.30 ± 0.47	0.29 ± 0.47	0.989
Breathiness	0.07 ± 0.25	0.10 ± 0.32	0.005
Asthenia	0.14 ± 0.36	1.16 ± 0.63	0.002
Strain	0.11 ± 0.31	0.14 ± 0.39	0.036
Acoustic metrics			
Maximum phonation time (MPT, s)	6.76±5.20	7.36±5.43	0.016
Fundamental frequency (F0, Hz)	171.79±63.14	173.93±71.66	0.493
Jitter (%)	0.62±0.49	0.60±0.44	0.468
Shimmer (%)	2.36±1.52	2.14±1.32	0.001
Harmonic noise ratio (HNR, dB)	17.72 ± 4.472	17.81 ± 4.18	0.642

† Continuous variables were presented as mean ± standard deviation (SD) and compared using independent Student's t-test. Categorical variables were presented as percentage (ratio) and compared using the Pearson chi-square test.

Table S9. Efficacy of best performing classification models on the external validation set.

	model	Class[§]	Accuracy	Specificity	ROC AUC	Sensitivity	Recall	F1 score
multiclass	Neural network	0	0.779	0.591	0.912	0.967	0.967	0.814
		1	0.932	0.977	0.923	0.611	0.611	0.692
		2	0.871	0.958	0.816	0.256	0.256	0.331
		3	0.876	0.984	0.774	0.122	0.122	0.198
		4	0.900	0.946	0.873	0.577	0.578	0.591
binary	CatBoost	-	0.854	0.8472	0.909	0.8611	0.876	0.855

[§] Class labels: 0, no dysphagia; 1, oral phase dysphagia; 2, pharyngeal phase dysphagia; 3, esophageal phase dysphagia; 4, oropharyngeal dysphagia.

Table S10. Parameter names and value ranges for machine learning grid search.

Machine Learning Algorithm	Hyperparameter	Search Range	Best Value (Multi)	Best Value (Binary)
Logistic Regression	C	0.1, 1, 10	1	1
	solver	'liblinear', 'lbfgs'	'liblinear'	'lbfgs'
	penalty	'l1', 'l2'	l2	l2
K-Nearest Neighbors	n_neighbors	3, 5, 7	5	5
	weights	'uniform', 'distance'	'distance'	'distance'
	P	1, 2	1	1
Support Vector Machine	kernel	'Linear', 'RBF'	'RBF'	'RBF'
	C	0.1, 1, 10	1	10
	gamma	'scale', 'auto'	'auto'	'scale'
Random Forest	n_estimators	100, 200	100	200
	max_depth	None, 10, 20	10	10
	max_features	'sqrt', 'log2'	'sqrt'	'sqrt'
	bootstrap	'True', 'False'	'False'	'False'
Gradient Boosting Machine	learning_rate	0.01, 0.05, 0.1	0.05	0.05
	n_estimators	50, 100, 200	100	50
	max_depth	3, 5	3	5
XGBoost	learning_rate	0.01, 0.05, 0.1	0.05	0.05
	max_depth	3, 6, 9	6	6
	n_estimators	50, 100	50	50
LightGBM	learning_rate	0.01, 0.05, 0.1	0.05	0.05
	n_estimators	50, 100	100	100
	max_depth	5, 6,	5	5
CatBoost	learning_rate	0.01, 0.05, 0.1	0.1	0.1
	depth	4, 6	4	6
	iterations	50, 100	100	100
Neural Network	activation	'relu', 'tanh'	'tanh'	'relu'
	hidden_layer_sizes	50, 100	100	100
	alpha	0.0001, 0.001	0.001	0.0001
